# Attitudes towards vaccination are associated with vaccination behaviour among university students: Jannis Trümmler

**DOI:** 10.1093/eurpub/ckac130.019

**Published:** 2022-10-25

**Authors:** J Trümmler, E Heumann, SM Helmer, C Stock, H Busse, K Heinrichs, S Negash, J Horn, Y Niephaus, CR Pischke

**Affiliations:** 1 Institute of Medical Sociology, Heinrich Heine University Duesseldorf, Duesseldorf, Germany; 2 Institute of Health and Nursing Science, Charité-Universitätsmedizin Berlin, Berlin, Germany; 3 Unit of Health Promotion Research, University of Southern Denmark, Esbjerg, Denmark; 4 Department Prevention and Evaluation, Institute for Prevention Research and Epidemiology, Bremen, Germany; 5 Institute for Medical Epidemiology, Martin-Luther University Halle-Wittenberg, Halle (Saale), Germany; 6 Department of Social Sciences, University of Siegen, Siegen, Germany

## Abstract

**Introduction:**

Vaccination behaviour is influenced by various determinants. Evidence indicates a higher COVID-19 vaccine hesitancy among university students due to their age and a lower risk of complications compared to the general population in Germany. However, little is known about other COVID-19-related determinants for the population of German university students. This study aimed to investigate determinants of vaccination behaviour among German university students.

**Methods:**

The cross-sectional COVID-19 German Student Well-being Study was conducted at five German universities at the end of 2021 via an online survey (n = 7.267). Multiple logistic regressions were calculated to examine associations of vaccination behaviour (not vaccinated vs. fully vaccinated) and attitudes towards vaccination (5Cs: confidence in the safety of the vaccine, complacency - not perceiving diseases as high risk, constraints - structural and psychological barriers, calculation - engagement in information seeking, collective responsibility - willingness to protect others), health literacy in a pandemic (CHL-P), and additional determinants.

**Results:**

All 5Cs were associated with the vaccination status ‘fully vaccinated’, except for complacency. Regarding CHL-P, we found that students who felt that the current scientific knowledge about COVID-19 in terms of the policy decisions on pandemic measures was very complex had a higher odds for being vaccinated (OR = 3.02; 95% CI: 2.26-4.04). Regarding additional determinants, the analysis revealed that students who had been previously infected had in all regressions a lower odds for being vaccinated compared to students with no previous infection.

**Conclusions:**

Due to the strong association of the attitudes towards vaccination and vaccination behaviour among university students, we recommend that the different components of the 5Cs should be considered in future COVID-19 vaccination campaigns in the university context.

**Key messages:**

- Attitudes towards vaccinations impact on vaccination behaviour among university students.
- Perceiving the current scientific knowledge on COVID-19 as very complex increased the odds for vaccination.

